# Epidemiological, Clinicopathological, and Surgical Characteristics of Eyelid Carcinomas: A Three-Year Retrospective Case Series From Eastern Morocco

**DOI:** 10.7759/cureus.113507

**Published:** 2026-07-28

**Authors:** Soufyane Elkadiri, Ismail Hailouma, Doha Arreyouchi, Ayat Allah Oufkir

**Affiliations:** 1 Plastic and Reconstructive Surgery, Mohammed VI University Hospital, Oujda, MAR; 2 Research Laboratory in Medical Sciences, Faculty of Medicine and Pharmacy, Mohamed I University, Oujda, MAR

**Keywords:** basal cell carcinoma, biopsy, carcinomas, eyelids, flaps and grafts, reconstruction, surgery

## Abstract

Background

Eyelid carcinomas are the most common malignant tumors of the periocular region and require a balance between complete oncologic resection and functional reconstruction. This study aimed to describe the demographic, clinical, and histopathological characteristics of patients with eyelid carcinoma treated surgically at a tertiary referral center in Eastern Morocco, while evaluating surgical management strategies, reconstructive approaches, postoperative complications, and short- to mid-term recurrence outcomes.

Methods

This retrospective study was conducted over a three-year period, from January 2023 to December 2025, and included 20 patients with eyelid carcinomas who underwent surgical treatment in the Department of Plastic and Reconstructive Surgery at Mohammed VI University Hospital, Oujda, Morocco. Demographic, clinical, histopathological, therapeutic, and outcome data were analyzed.

Results

Twenty patients were included, comprising 11 women (55%) and 9 men (45%), with a mean age of 63.1 years. Chronic sun exposure was identified in 18 patients (90%). The lower eyelid was the most commonly affected site in nine cases (45%). Basal cell carcinoma (BCC) accounted for 15 tumors (75%), followed by squamous cell carcinoma (SCC) in five cases (25%). Wide local excision with histopathological margin control was performed in all patients (20/20, 100%), and orbital exenteration was required in one case (5%). Reconstruction was achieved mainly using local flaps in 12 patients (60%). Ectropion occurred in two patients (10%). No local recurrence was observed during a mean follow-up of 22 months.

Conclusion

Eyelid carcinomas, particularly BCC, represent a significant therapeutic challenge, with surgery remaining the mainstay of treatment. The optimal management strategy relies on prevention and early diagnosis of lesions, which are essential to improve prognosis and prevent disease progression.

## Introduction

Eyelid carcinomas are malignant tumors of the periocular region, with basal cell carcinoma (BCC) accounting for approximately 90% of cases, followed less frequently by squamous cell carcinoma (SCC) and sebaceous carcinoma. The incidence and histological distribution vary according to geographic location, ultraviolet (UV) exposure, and population skin phototype [1,2].

Early diagnosis remains the cornerstone of effective management and is based on clinical examination, biopsy with histopathological confirmation, and appropriate imaging when orbital extension is suspected. The therapeutic challenge lies in achieving complete tumor excision while preserving eyelid function, ocular protection, and satisfactory aesthetic outcomes [3].

To our knowledge, data on the epidemiological, clinicopathological, and management characteristics of eyelid carcinomas in Morocco, particularly in the eastern region, which is characterized by high levels of sunshine and UV exposure, remain scarce. This study was therefore undertaken to describe the epidemiological and clinicopathological characteristics of eyelid carcinomas and to evaluate the surgical management of patients treated at our institution, providing regional data that may contribute to a better understanding of the disease and support future clinical research.

## Materials and methods

This retrospective descriptive study was conducted in the Department of Burn, Plastic, Reconstructive, and Aesthetic Surgery at Mohammed VI University Hospital in Oujda, Morocco, a tertiary referral center, over a three-year period from January 2023 to December 2025.

We included all patients with histologically confirmed eyelid carcinoma who underwent surgical treatment within the Department of Burn, Plastic, Reconstructive, and Aesthetic Surgery during the study period. Patients managed exclusively with non-surgical approaches, as well as those with incomplete essential clinical data or without pathological confirmation of malignancy, were excluded. Patients with incomplete postoperative follow-up were not excluded from the study population; however, they were identified separately and considered in the outcome analysis. Duplicate records were carefully reviewed and removed to prevent double-counting.

Data were collected from hospital medical records and operative logs by the authors using a standardized review process covering clinical, operative, pathological, and follow-up data. The following variables were analyzed: age, sex, skin phototype, risk factors, delay to consultation, tumor location, clinical presentation, tumor size, histopathological diagnosis, imaging findings, surgical management, reconstructive technique, postoperative complications, and follow-up outcomes.

Clinical assessment included evaluation of tumor morphology, involved eyelid subunits, and regional lymph node status. Histopathological diagnosis was established by preoperative biopsy and confirmed on the surgical excision specimen. Orbital computed tomography (CT) and/or magnetic resonance imaging (MRI) were performed in selected patients when deep tissue or orbital invasion was suspected clinically.

Preoperative and postoperative clinical photographs were obtained for documentation and postoperative morphological assessment of the reconstructive outcomes.

Statistical analysis was performed using Microsoft Excel (Microsoft Corporation, Redmond, WA, USA). Continuous variables were expressed as mean ± standard deviation and range, whereas categorical variables were reported as frequencies and percentages. Given the limited sample size, no inferential statistical analyses were performed.

This study was conducted in accordance with institutional and national ethical standards. Given the retrospective design of the study and the use of anonymized patient data, formal approval from the institutional review board/ethics committee was not required according to local regulations. All patient data were handled in a de-identified manner to ensure confidentiality. Furthermore, all patients routinely provided written informed consent prior to surgery, including authorization for the use of their clinical photographs for scientific and educational purposes, including publication in peer-reviewed journals.

## Results

Patient characteristics and risk factors

Twenty patients were included in the study: 11 women (55%) and 9 men (45%). The mean age at diagnosis was 63.1 ± 22.3 years (range, 16-90 years).

Regarding skin phototype, five patients (25%) had Fitzpatrick phototype II, nine patients (45%) had phototype III, and six patients (30%) had phototype IV [4]. Most patients reported substantial lifetime sun exposure, often related to outdoor occupational activities such as farming or security work. Overall, chronic sun exposure was identified in 18 patients (90%). The remaining two patients (10%) had limited cumulative sun exposure due to their young age; however, they remained highly susceptible to UV radiation because of a history of xeroderma pigmentosum.

Actinic keratosis was noted in one patient (5%), a history of eyelid trauma in one patient (5%), and chronic tobacco use in two patients (10%), both of whom were male. Xeroderma pigmentosum was present in two patients (10%). No history of arsenic exposure or prior radiotherapy was documented in this series. The delay between lesion onset and consultation ranged from 2 to 60 months, with a mean of 34 ± 18.2 months.

Tumor location and clinical presentation

The lower eyelid was the most frequently involved anatomical site, followed by the medial canthus. Upper eyelid involvement, lateral canthal lesions, and tumors affecting multiple subunits were less common (Table 1).

**Table 1:** Anatomical distribution of lesions according to eyelid aesthetic subunits (N = 20).

Anatomical site	Number of cases (N)	Percentage %
Lower eyelid	9	45
Medial canthus	3	15
Upper eyelid	2	10
Lateral canthus	2	10
Multiple subunits involvement	4	20

The nodular form was the most common clinical presentation, followed by ulcerated lesions and exophytic or ulcerative-exophytic tumors. Tumor size ranged from 4 mm to 6 cm along the greatest axis (Table 2).

**Table 2:** Clinical morphological patterns of lesions (N = 20).

Morphological presentation	Number of cases (N)	Percentage (%)
Nodular form	8	40
Ulcerated form	7	35
Exophytic/ulcerative-exophytic	5	25

Histopathology and imaging findings

Preoperative biopsy was performed in all patients. Histopathological examination revealed 15 cases of BCC (75%), distributed as follows: nine nodular (45%), three superficial (15%), and three sclerodermiform (15%) (morpheaform) subtypes.

The remaining five cases (25%) were well-differentiated, infiltrating, keratinizing SCC (Table 3).

**Table 3:** Clinicopathological and AJCC staging characteristics of patients with squamous cell carcinoma (SCC) (n = 5). * TNM classification according to the AJCC Cancer Staging Manual, 8th edition [5]. Abbreviations: AJCC, American Joint Committee on Cancer; SCC, squamous cell carcinoma; R0, histologically negative surgical margins

Cases	Tumor size (mm)	TNM classification*	AJCC stage	Depth of invasion	Surgical margins (≥ 6 mm)	Clinical and radiological assessment
1	8	pT1 cN0 cM0	I	No involvement of the tarsal plate or eyelid margin	R0	cN0
2	5	pT1 cN0 cM0	I	No involvement of the tarsal plate or eyelid margin	R0	cN0
3	6	pT1 cN0 cM0	I	No involvement of the tarsal plate or eyelid margin	R0	cN0
4	17	pT2b cN0 cM0	IIA	Eyelid margin involvement	R0	cN0
5 (Orbital exenteration case)	22	pT4a cN0 cM0	IIB	Orbital invasion	R0	cN0

No evidence of perineural invasion or lymphovascular invasion was identified on histopathological analysis. Furthermore, no discordance was observed between the preoperative biopsy findings and the final histopathological diagnosis of the excision specimens.

Orbital CT was performed in four patients (20%) because of tumor size, fixation, or suspected local invasion on clinical examination. In two cases (10%), CT showed no involvement of adjacent structures. Periorbital soft-tissue invasion was identified in one patient (5%). In another patient (5%), the tumor was adherent to the orbicularis muscle and the zygomatic bone without evidence of bone erosion.

Orbital MRI was obtained in one patient and demonstrated a left preorbital ulcerative exophytic mass extending to the ocular globe, adjacent osseous structures, and preseptal orbital fat.

Surgical management and reconstruction

Mohs micrographic surgery was not available in our institution. Therefore, the standard management strategy consisted of wide local excision with safety margins ranging from 6 mm to 1 cm, followed by delayed eyelid reconstruction after confirmation of tumor-free margins on permanent paraffin-embedded sections. Reconstruction was generally performed approximately one week after tumor excision, once the final pathology report confirmed complete tumor excision. Orbital exenteration was required in one patient because of extensive local invasion.

All excision specimens were systematically submitted for routine histopathological examination to confirm the diagnosis and assess surgical margin status. Intraoperative frozen-section analysis was not available during the study period. Consequently, surgical defects were temporarily managed with appropriate dressings, and reconstruction was deferred until the final pathology results were available. Negative surgical margins were confirmed in all cases before reconstruction.

**Figure 1:**
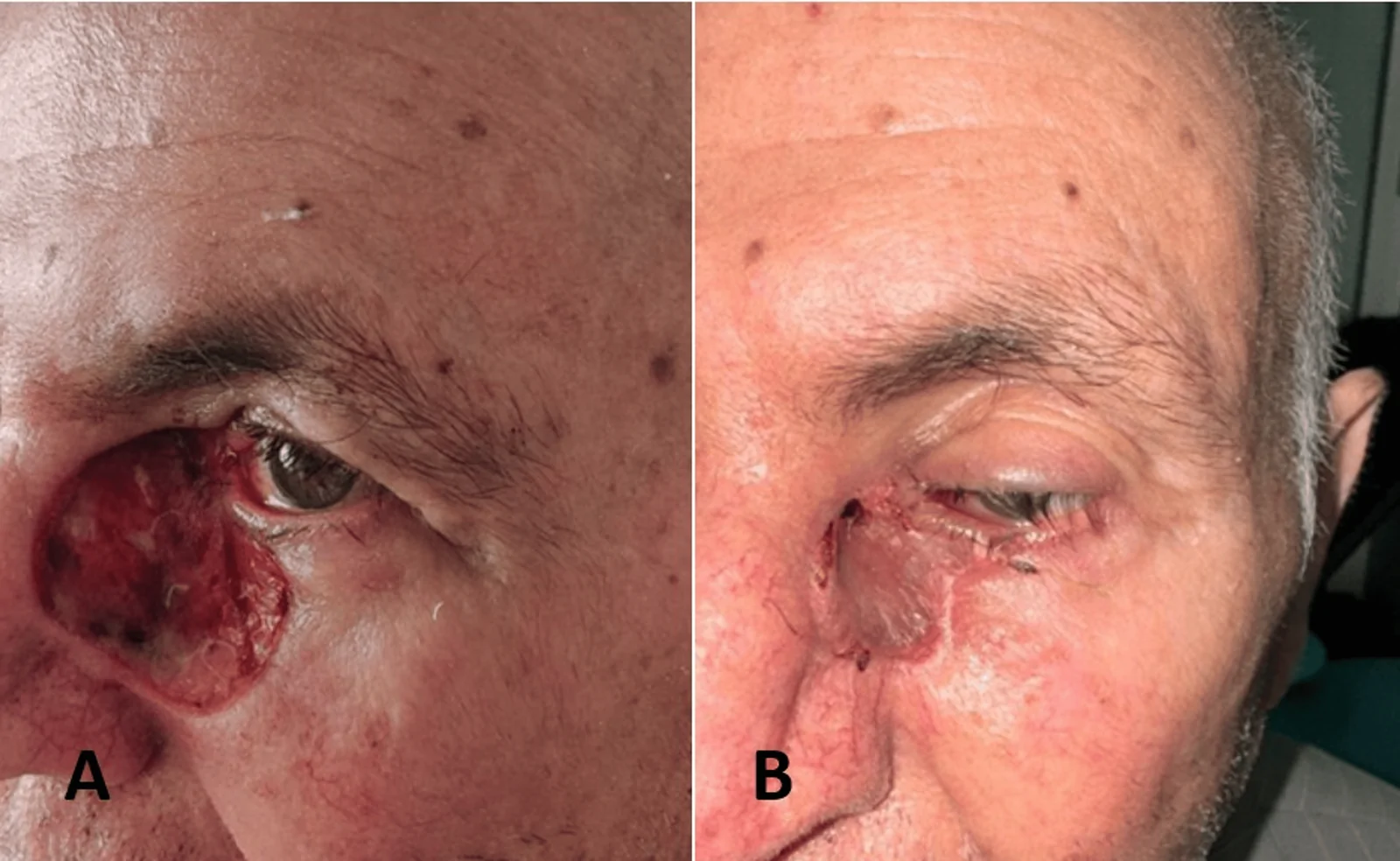
(A) Post-excision defect following resection of a basal cell carcinoma of the medial canthus. (B) Fifteen days postoperative outcome showing satisfactory graft take. The patient provided written and signed consent, allowing publication of this identifiable facial image in an open-access journal.

Reconstruction was individualized according to defect size, location, and involvement of the anterior and posterior lamellae. Direct closure was used in four patients (20%), secondary intention healing in one patient (5%), full-thickness skin grafting in three patients (15%) (Figure 1), and local flaps in 12 patients (60%) (Table 4).

**Table 4:** Reconstructive techniques used for eyelid defect reconstruction (N = 20).

Reconstructive technique	Number of cases (N)	Percentage (%)
Direct closure	4	20
Secondary intention healing	1	5
Full-thickness skin graft	3	15
Local flaps	12	60

**Figure 2:**
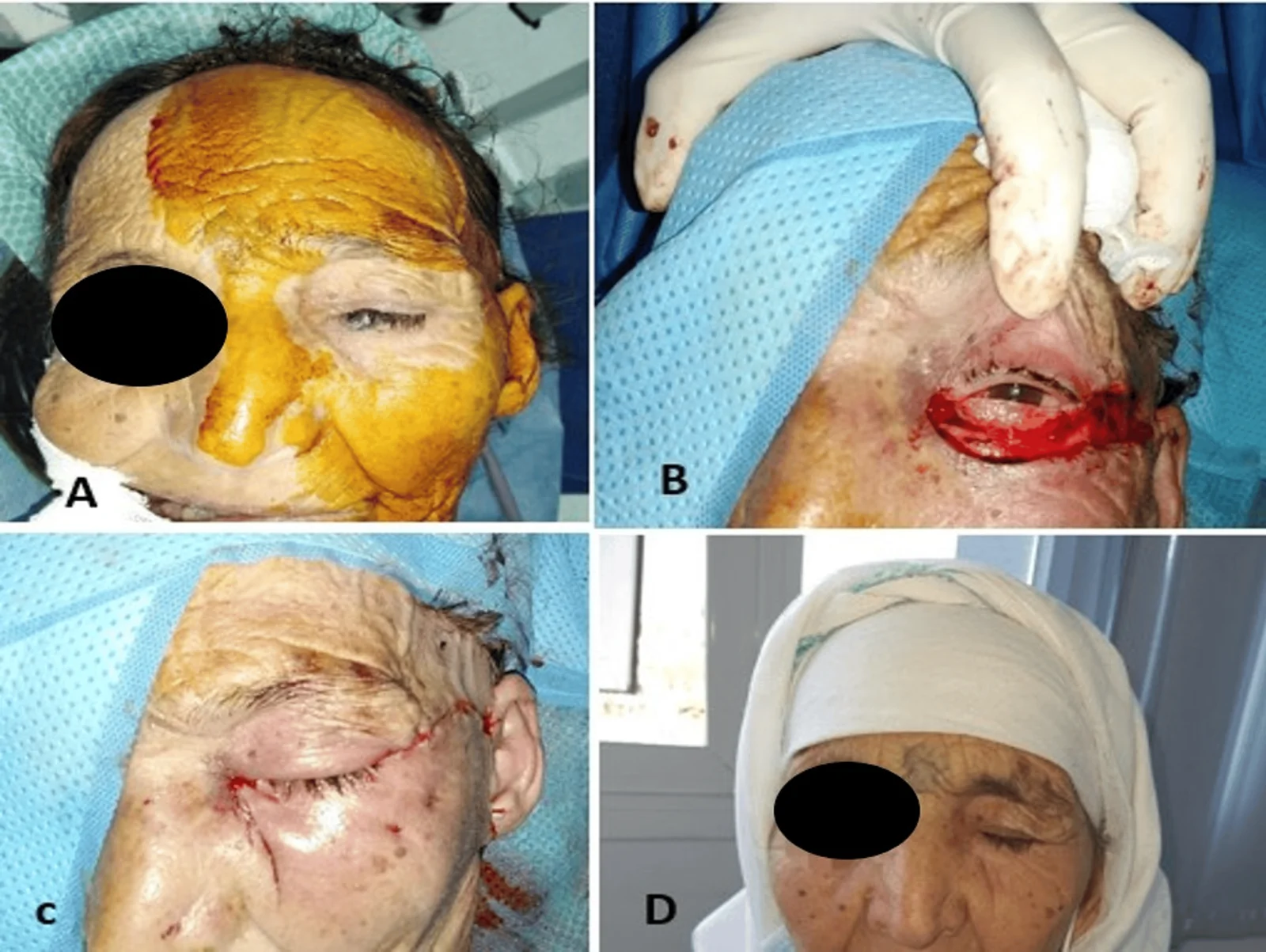
(A) Preoperative appearance of a nodular basal cell carcinoma of the lower eyelid. (B) Immediate post-excision defect. (C) Intraoperative view of the Mustardé flap. (D) Frontal postoperative view at three months. The patient provided written and signed consent, allowing publication of this identifiable facial image in an open-access journal.

**Figure 3:**
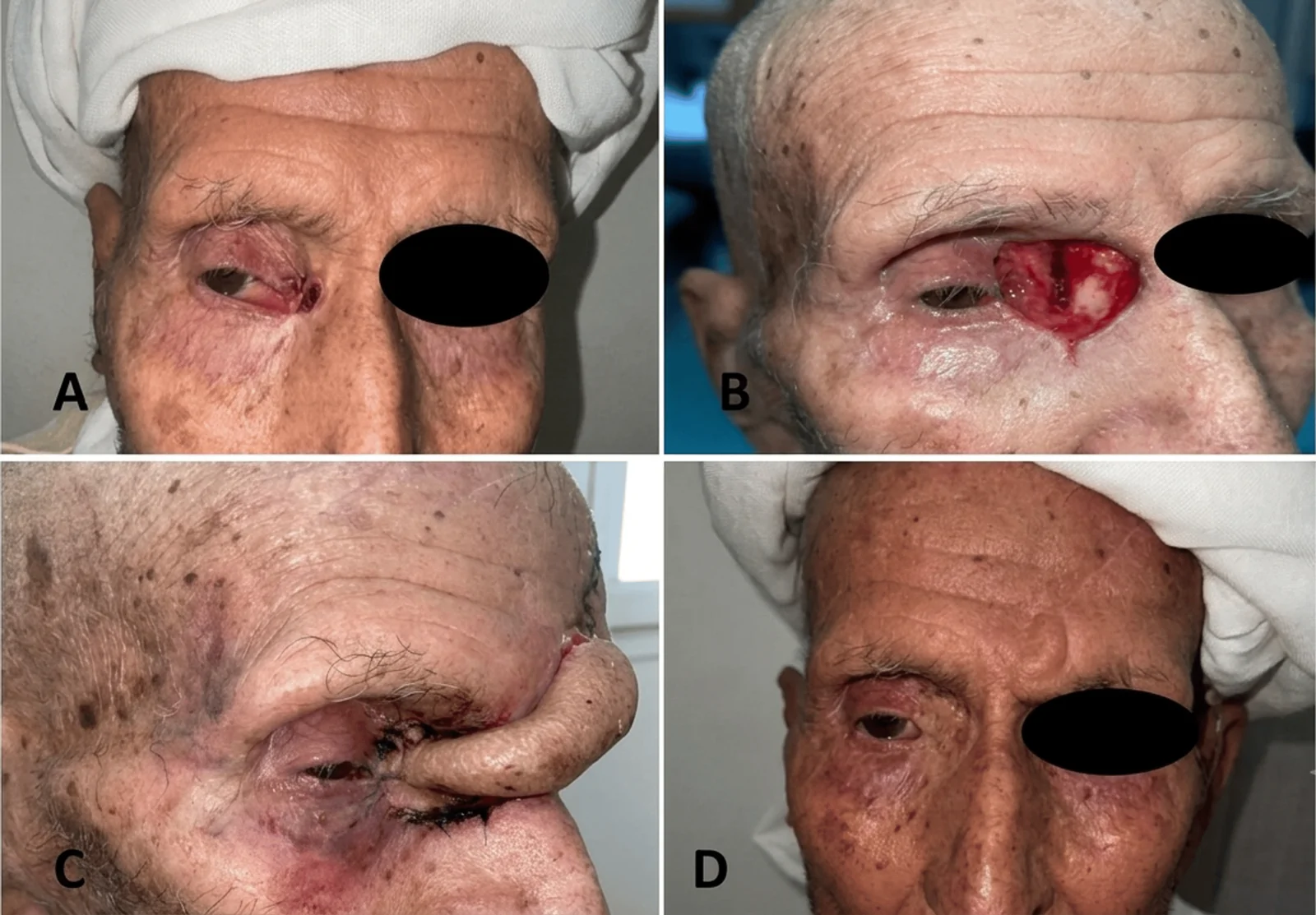
(A) Nodular basal cell carcinoma of the medial canthus. (B) Immediate post-excision defect. (C) Paramedian forehead flap. (D) Postoperative outcome at three months. The patient provided written and signed consent, allowing publication of this identifiable facial image in an open-access journal.

**Figure 4:**
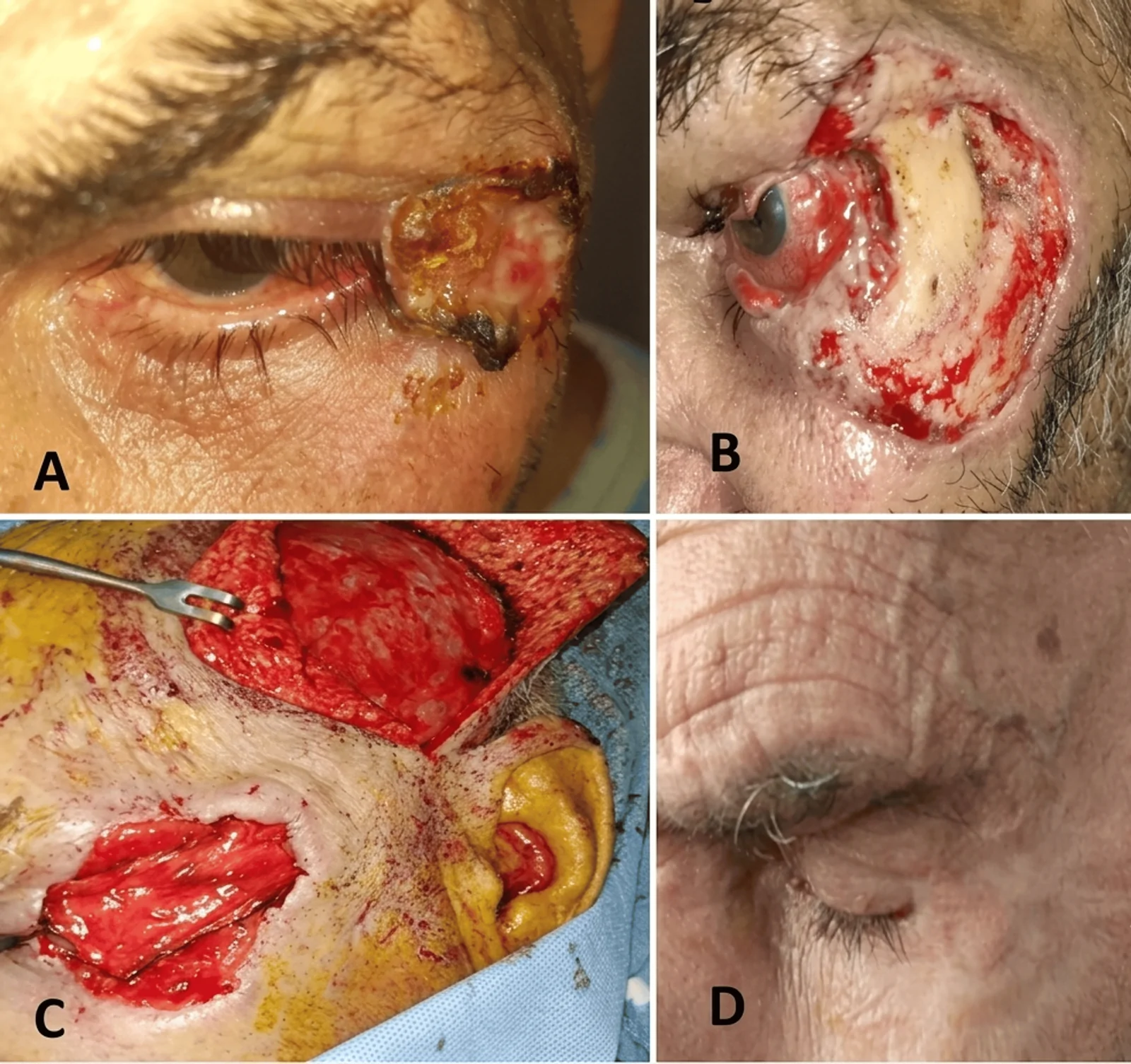
(A) Squamous cell carcinoma of the lateral canthus. (B) Post-excision defect with exposure of the zygomatic bone. (C) Reconstruction using a superficial temporal fascia flap with skin grafting. (D) Postoperative outcome at 12 months. The patient provided written and signed consent, allowing publication of this identifiable facial image in an open-access journal.

**Figure 5:**
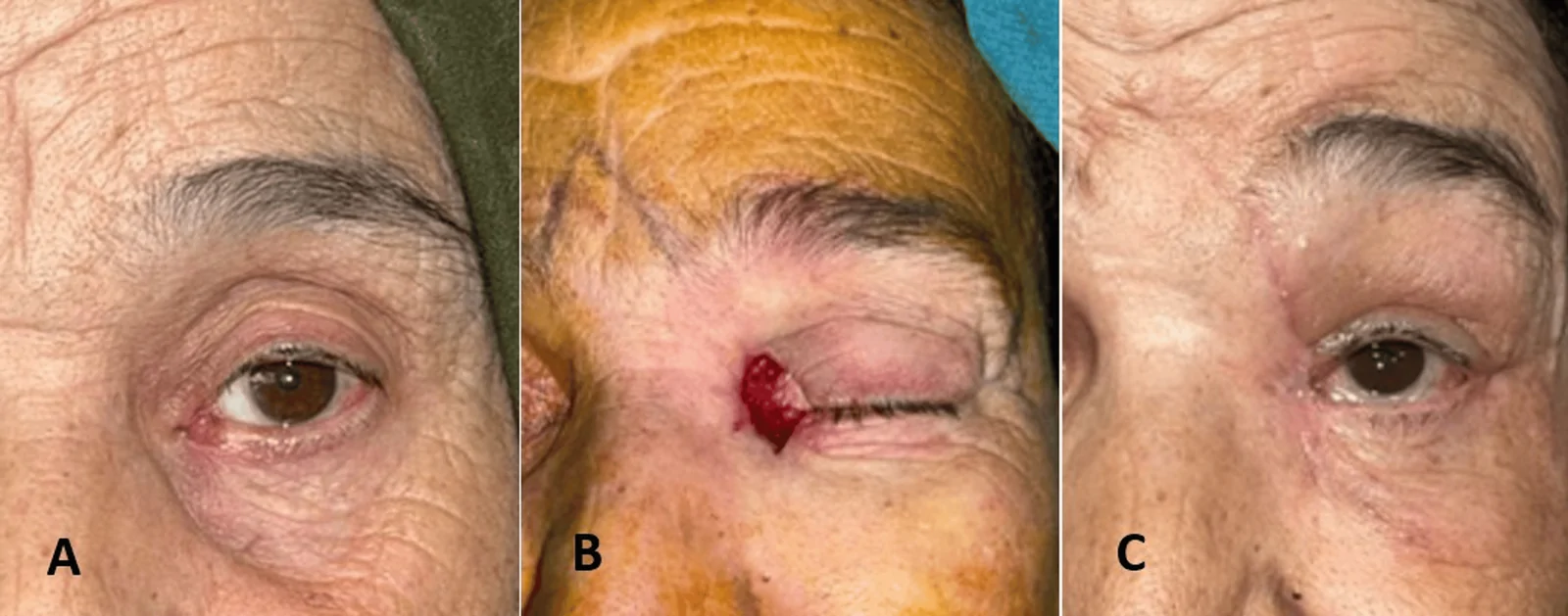
(A) Basal cell carcinoma of the lower eyelid. (B) Design of a glabellar flap. (C) Postoperative outcome at three months. The patient provided written and signed consent, allowing publication of this identifiable facial image in an open-access journal.

Among flap-based reconstructions, the Mustardé temporojugal flap was the most frequently used technique (Figure 2). Other flap procedures included paramedian forehead flaps (Figure 3), superficial temporal fascia-based pedicled flaps (Figure 4), and glabellar flaps (Figure 5 and Table 5).

**Table 5:** Distribution of local flap techniques used for eyelid reconstruction (N = 12).

Flap techniques	Number of cases (N)	Percentage (%)
Mustardé temporo-jugal	5	41.7
Forehead	3	25
Superficial temporal fascia-based pedicled	2	16.7
Glabellar	2	16.7

The Mustardé temporojugal flap was combined with a palatal fibromucosal graft in four cases (20%) and with a nasal chondromucosal graft in one case (5%) to reconstruct the posterior lamella.

Postoperative outcomes

**Figure 6:**
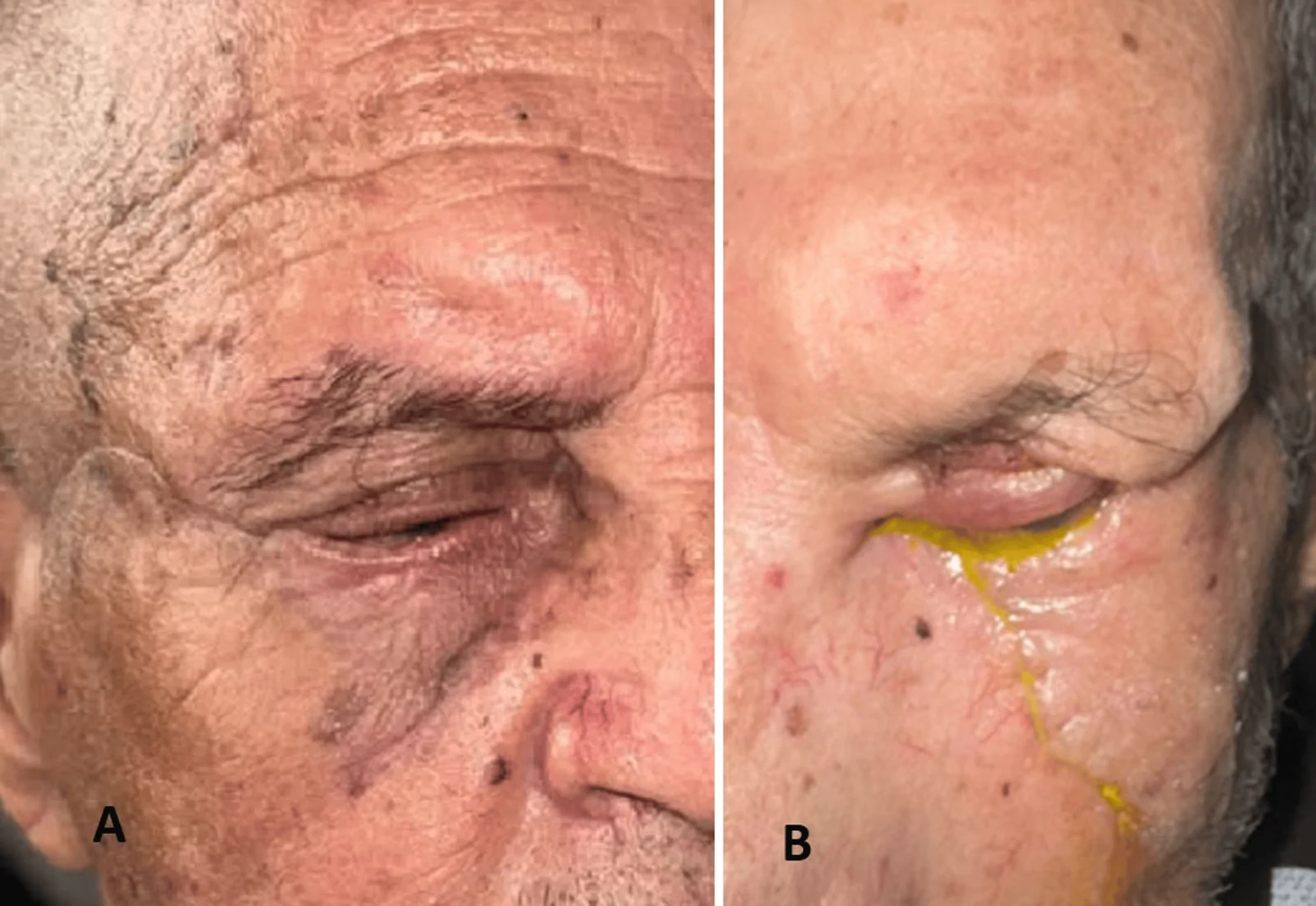
Postoperative outcomes: (A) ectropion, (B) ectropion associated with bacterial conjunctivitis. The patient provided written and signed consent, allowing publication of this identifiable facial image in an open-access journal.

Postoperative ectropion occurred in two patients (10%), including one case associated with bacterial conjunctivitis (Figure 6). Both patients underwent revision surgery to correct the eyelid malposition. The patient with bacterial conjunctivitis also received appropriate antibiotic therapy, resulting in complete resolution of the infection. No flap or graft failure was observed during the postoperative period, and all reconstructions remained viable throughout the available follow-up.

In the remaining patients, postoperative clinical evaluation demonstrated complete eyelid closure without lagophthalmos, adequate corneal protection, an adequate palpebral fissure opening, and preserved blink function. No cases of exposure keratopathy or persistent ocular irritation were documented during follow-up. Cosmetic evaluation showed preserved symmetry with the contralateral eyelid, appropriate eyelid margin position, and satisfactory scar appearance in most patients. Apart from the two patients who required revision surgery for ectropion, no additional secondary corrective procedures were performed.

Follow-up data were available for 18 patients (90%), with a mean follow-up duration of 22 months (range, 6-36 months). No local recurrence was observed during the available follow-up period. Postoperative clinical evaluation suggested stable functional and cosmetic outcomes in the patients who completed follow-up. However, these findings were based on descriptive clinical assessment rather than standardized functional or aesthetic outcome measures. Furthermore, the limited number of patients with available follow-up and the relatively short follow-up duration in some cases should be considered when interpreting these findings.

## Discussion

Eyelid carcinomas, particularly BCC, show a clear geographical variation that is strongly influenced by UV radiation exposure and population skin phototype. The highest incidence rates are reported in regions with high ambient UV exposure, such as Australia and parts of Southern Europe, while lower rates are generally observed in more northern latitudes. This distribution is also closely linked to the proportion of fair-skinned populations who are more susceptible to UV-induced DNA damage. In contrast, in African and darker-skinned populations, eyelid carcinomas are less frequent but often diagnosed at a later stage, likely due to reduced clinical suspicion and healthcare access disparities. Overall, geographic differences reflect a combination of environmental UV exposure, genetic susceptibility, and healthcare-related factors [6]. In the Moroccan context, particularly in the eastern region, eyelid carcinomas may present specific diagnostic and therapeutic challenges. The high level of annual sunshine and substantial occupational UV exposure among outdoor workers may contribute to the development of UV-related eyelid malignancies.

In the present series, the mean age at diagnosis was 63.1 years, which is consistent with previously published reports showing that eyelid malignancies predominantly affect older adults [3,7-9]. However, the occurrence of carcinoma in two young patients with xeroderma pigmentosum highlights the major role of genetic susceptibility in early-onset cutaneous malignancy (Table 6).

**Table 6:** Comparative analysis of mean age and age range in patients with eyelid tumors.

Authors	Study year	Number of cases	Mean age (years)	Age range (years)
Echchaoui et al. [3]	2009-2013	64	60	33-93
Ul Kadir et al. [7]	2014-2019	332	62.4	34-96
Kaliki et al. [8]	1995-2016	536	60	4-100
Yu et al. [9]	2002-2015	2228	67	1-98
Present study	2023-2025	20	63.1	16-90

In our cohort, phototypes III and IV were predominant, reflecting the skin phototype distribution commonly encountered in the Moroccan population [3]. By contrast, studies from Brazil [10] and France [11] have described a predominance of lighter skin phototypes; these differences likely reflect geographic and ethnic variations in the distribution of skin phototypes (Table 7). Nevertheless, chronic sun exposure remained the most frequent risk factor, being identified in 90% of patients. This finding is in line with the well-established role of cumulative UV exposure in the pathogenesis of nonmelanoma skin cancers, particularly in individuals engaged in outdoor occupations. The prolonged mean delay to consultation in our series (34 months) is also noteworthy and likely contributed to the locally advanced presentation observed in some patients. Similar delays have been reported in other regional studies and may reflect limited awareness, underestimation of lesion severity, and delayed access to specialized care [3,12].

**Table 7:** Comparison of Fitzpatrick skin phototype profiles across studies.

Study	Predominant phototypes	Percentage (%)
Echchaoui et al. [3]	III and IV	78.1
Ferreira et al. [10]	II and III	77
Dumas et al. [11]	II and III	100
Present study	III and IV	75

The lower eyelid was the most frequently involved site, followed by the medial canthus, which is consistent with most published series [3,13-15]. This distribution is generally attributed to greater cumulative UV exposure of the lower eyelid and the anatomical vulnerability of the periocular region (Table 8).

**Table 8:** Distribution of anatomical sites of eyelid carcinomas.

Series	Lower eyelid (%)	Upper eyelid (%)		Medial canthus (%)	Lateral canthus (%)
Echchaoui et al. [3]	53	10		31	6
Older et al. [13]	53	12		27	8
Thiagarajan et al. [14]	53	35		6	6
Poignet et al. [15]	76.6	9.4		7	2.3
Present study	45	10		15	10

A diagnostic biopsy is first performed to establish the histological nature of the lesion and to guide the choice of appropriate management based on the results [13]. On histopathological analysis, BCC was the predominant subtype in our study, accounting for 75% of cases, followed by SCC (25%). This pattern is in agreement with the literature, in which BCC consistently represents the most common eyelid malignancy [2]. Although less frequent, SCC remains clinically important because of its more aggressive behavior and greater metastatic potential.

Imaging played a selective but important role in our series. CT and MRI were reserved for patients with large, fixed, or clinically invasive tumors to evaluate deep extension, orbital involvement, and possible bone invasion. This approach is consistent with current practice, as cross-sectional imaging is particularly useful in locally advanced lesions and contributes to both staging and surgical planning [16,17].

Surgical excision remains the cornerstone of treatment for eyelid carcinomas. When margin involvement is identified, a secondary procedure with wider excision is recommended to achieve histologically clear margins and reduce the risk of local recurrence [2]. In this context, techniques such as frozen-section margin control or Mohs micrographic surgery can further optimize oncologic outcomes by enabling real-time assessment of surgical margins [18]. In the absence of Mohs micrographic surgery, surgical margins were determined according to the recommendations of the National Comprehensive Cancer Network (NCCN). Radiotherapy represents an alternative or adjunctive treatment modality in selected cases, particularly for patients who are not candidates for surgery, those with perineural invasion, or those with positive surgical margins when further excision is not feasible. It may also be used as adjuvant therapy to improve local control [19,20].

Eyelid reconstruction aims to restore both functional integrity and aesthetic appearance of the eyelid. The choice of the technique was based on several factors, including the size, location, and depth of the defect. In our setting, reconstruction is generally performed in a delayed manner because intraoperative frozen-section analysis is not routinely available, and logistical constraints may further prolong anesthesia time. The reconstructive approach was individualized according to the size, location, and depth of each defect. In this series, posterior lamella reconstruction (tarsoconjunctival layer) was primarily achieved using palatal mucosal grafts (20% of cases), owing to their appropriate curvature and satisfactory rigidity [21]. For anterior lamella reconstruction (cutaneous-muscular layer), the most frequently employed techniques were direct closure and local flaps, including the Mustardé temporojugal flap, the forehead flap, and the pedicled glabellar flap. This predominance can be explained by the high frequency of tumor involvement of the lower eyelid and medial canthal region, for which these flaps provide particularly suitable reconstructive options [22]. For reconstruction of exenteration cavities, we used pedicled superficial fascia flaps combined with full-thickness skin grafts, ensuring reliable cutaneous coverage and satisfactory healing.

The main complications observed were ectropion and bacterial conjunctivitis. These findings highlight the critical importance of adequate posterior lamella reconstruction, particularly in full-thickness eyelid defects, as well as the need for appropriate local antibiotic prophylaxis.

The management of eyelid carcinomas in Eastern Morocco may involve specific challenges that can influence both diagnosis and therapeutic strategies. Delayed presentation may contribute to the diagnosis of larger and more advanced lesions, which often require more extensive surgical excision and complex reconstructive procedures. In addition, significant occupational exposure to UV radiation, particularly among outdoor workers, may represent an important contributing factor to the occurrence of eyelid malignancies in this population. Furthermore, access to specialized diagnostic and therapeutic resources, such as Mohs micrographic surgery and intraoperative frozen-section analysis, remains limited in some healthcare settings. Consequently, treatment decisions are frequently based on careful clinical assessment, conventional histopathological examination, and the expertise of the surgical team. These contextual factors highlight the importance of epidemiological studies from our region and underscore the need for improved prevention strategies, early detection programs, and the development of specialized resources for the management of eyelid cancers.

This study has several limitations. First, its retrospective design may have introduced selection and information biases. Second, the small sample size limits the statistical power and generalizability of the findings. Third, the single-center design may not fully reflect the epidemiological characteristics of eyelid carcinomas in the general population.

Although favorable outcomes were observed after surgical excision with individualized reconstruction, these results should be interpreted cautiously due to the limited sample size and follow-up duration. The absence of recurrence in our cohort does not necessarily reflect long-term oncological control. Larger prospective multicenter studies with longer follow-up are needed to confirm these findings and better define prognostic factors and optimal management strategies.

## Conclusions

Eyelid carcinomas are uncommon but clinically significant malignancies that predominantly affect older adults and are strongly associated with cumulative UV radiation exposure, with BCC representing the most frequent histological subtype. Their management remains challenging, requiring a careful balance between complete oncological excision and preservation of eyelid function and aesthetics. Early diagnosis and timely referral are essential to improving outcomes and reducing the morbidity associated with advanced disease. Complete surgical excision, combined with individualized reconstructive strategies tailored to the size, location, and depth of the defect, remains the cornerstone of successful management, providing satisfactory functional and cosmetic outcomes. In settings characterized by delayed presentation and limited access to specialized care, strengthening public awareness of early clinical signs and sun-protection measures while improving access to specialized diagnostic and therapeutic resources is crucial to reducing the burden of eyelid carcinomas and optimizing patient outcomes.

## References

[1] Coroi MC, Roşca E, Muţiu G (2010). Eyelid tumors: histopathological and clinical study performed in County Hospital of Oradea between 2000-2007. Rom J Morphol Embryol.

[2] Deprez M, Uffer S (2009). Clinicopathological features of eyelid skin tumors. A retrospective study of 5504 cases and review of literature. Am J Dermatopathol.

[3] Echchaoui A, Benyachou M, Houssa A (2016). Management of eyelid carcinomas: retrospective bicentric study of 64 cases and review of the literature (Article in French). J Fr Ophtalmol.

[4] Fitzpatrick TB (1988). The validity and practicality of sun-reactive skin types I through VI. Arch Dermatol.

[5] Sato Y, Takahashi S, Toshiyasu T, Tsuji H, Hanai N, Homma A (2024). Squamous cell carcinoma of the eyelid. Jpn J Clin Oncol.

[6] Rubin AI, Chen EH, Ratner D (2005). Basal-cell carcinoma. N Engl J Med.

[7] Ul Kadir SM, Rani Mitra M, Rashid R, Nuruddin M, Hassan Khan MK, Haider G, Nessa MS (2022). Clinicopathological analysis and surgical outcome of eyelid malignancies: a study of 332 cases. J Skin Cancer.

[8] Kaliki S, Bothra N, Bejjanki KM (2019). Malignant eyelid tumors in India: a study of 536 Asian Indian patients. Ocul Oncol Pathol.

[9] Yu SS, Zhao Y, Zhao H, Lin JY, Tang X (2018). A retrospective study of 2228 cases with eyelid tumors. Int J Ophthalmol.

[10] Ferreira FR, Nascimento LFC, Rotta O (2011). Risk factors for nonmelanoma skin cancer in Taubaté, São Paulo, Brazil: a case-control study (Article in Portuguese). Rev Assoc Med Bras.

[11] Dumas P, Benatar M, Cardot-Leccia N, Lebreton E, Chignon-Sicard B (2012). Study of skin retraction applied to the treatment of skin tumors. Mapping of the human body (Article in French). Ann Chir Plast Esthet.

[12] Chebbi A, Ben Amor R, Zghal I (2014). Basal cell carcinoma of the eyelids: diagnostic and therapeutic approach based on a series of 150 cases (Article in French). Research.

[13] Older JJ, Quickert MH, Beard C (1975). Surgical removal of basal cell carcinoma of the eyelids utilizing frozen section control. Trans Sect Ophthalmol Am Acad Ophthalmol Otolaryngol.

[14] Thiagarajan S, Bahani A, Chaukar D, Dcruz AK (2020). Eyelid carcinoma: an experience from a tertiary cancer center. J Cancer Res Ther.

[15] Poignet B, Gardrat S, Dendale R (2019). Basal cell carcinomas of the eyelid: results of an initial surgical management. J Fr Ophtalmol.

[16] Mafee MF, Rapoport M, Karimi A, Ansari SA, Shah J (2005). Orbital and ocular imaging using 3- and 1.5-T MR imaging systems. Neuroimaging Clin N Am.

[17] Purohit BS, Vargas MI, Ailianou A (2016). Orbital tumours and tumour-like lesions: exploring the armamentarium of multiparametric imaging. Insights Imaging.

[18] Lindgren G, Lindblom B, Bratel AT, Mölne L, Larkö O (2000). Mohs' micrographic surgery for basal cell carcinomas on the eyelids and medial canthal area. I. Characteristics of the tumours and details of the procedure. Acta Ophthalmol Scand.

[19] Schmults CD, Blitzblau R, Aasi SZ (2023). Basal cell skin cancer, version 2.2024, NCCN Clinical Practice Guidelines in Oncology. J Natl Compr Canc Netw.

[20] Schmults CD, Blitzblau R, Aasi SZ (2021). NCCN Guidelines® insights: squamous cell skin cancer, version 1.2022. J Natl Compr Canc Netw.

[21] Siegel RJ (1985). Palatal grafts for eyelid reconstruction. Plast Reconstr Surg.

[22] Yan Y, Fu R, Ji Q (2022). Surgical strategies for eyelid defect reconstruction: a review on principles and techniques. Ophthalmol Ther.

